# Cytosolic and mitochondrial Ca^2+^ signaling in procoagulant platelets

**DOI:** 10.1080/09537104.2021.1881951

**Published:** 2021-02-18

**Authors:** Sarah L. Millington-Burgess, Matthew T. Harper

**Affiliations:** Department of Pharmacology, University of Cambridge Cambridge, UK

**Keywords:** Thrombosis, Ca2+ signaling, coagulation, mitochondria

## Abstract

Platelets are the major cellular contributor to arterial thrombosis. However, activated platelets form two distinct subpopulations during thrombosis. Pro-aggregatory platelets aggregate to form the main body of the thrombus. In contrast, procoagulant platelets expose phosphatidylserine on their outer surface and promote thrombin generation. This apparently all-or-nothing segregation into subpopulations indicates that, during activation, platelets commit to becoming procoagulant or pro-aggregatory. Although the signaling pathways that control this commitment are not understood, distinct cytosolic and mitochondrial Ca^2+^ signals in different subpopulations are likely to be central. In this review, we discuss how these Ca^2+^ signals control procoagulant platelet formation and whether this process can be targeted pharmacologically to prevent arterial thrombosis.

## Introduction – a Procoagulant Platelet Subpopulation in Arterial Thrombosis

Platelets play a critical role in arterial thrombosis, a major cause of myocardial infarction, stroke, and ultimately death. However, current anti-platelet therapies carry increased bleeding risk, have substantial inter-individual variation, and may have lower and reduced effectiveness in some high-risk groups such as patients with diabetes [1,2]. These problems, and the continuing incidence of arterial thrombosis despite current anti-platelet strategies, indicate that new targets for thrombosis prevention are required.

During thrombosis, platelets adhere to exposed matrix proteins such as collagen fibers. These platelets activate and release soluble platelet activators that recruit further platelets[3]. Two distinct sub-populations of platelets form [4,5]. **Pro-aggregatory platelets** have active integrin α_IIb_β_3_, which allows them to bind fibrinogen and aggregate with other pro-aggregatory platelets to form the body of the thrombus. Current anti-platelet therapies reduce platelet aggregation, either by blocking the signals that lead to α_IIb_β_3_ activation, or by directly blocking α_IIb_β_3_[3]. In contrast, **procoagulant platelets** expose phosphatidylserine (PS), an efficient surface for assembly of coagulation complexes, generating a burst of thrombin that is responsible for producing an occlusive thrombus [6,7]. Procoagulant platelets also release PS-exposing extracellular vesicles (EVs), often called microparticles, that further promote coagulation [8,9]. Both pro-aggregatory and procoagulant platelets are essential to arterial thrombus growth[10]. Therefore, procoagulant platelets may be an alternative target to thrombosis prevention.

PS exposure is commonly monitored using annexin V, which binds to PS with high affinity in a Ca^2+^-dependent manner. Following stimulation with physiological activators, platelets segregate into two subpopulations, either with high annexin V binding or with binding no higher than unstimulated platelets [4,11–13]. The extent of high annexin V binding is similar to that seen with high concentrations of Ca^2+^ ionophores, which induce annexin V binding in all platelets. It is possible that a distinction based on annexin V obscures a more complex picture. Lactadherin can bind membranes with lower PS density in their outer leaflet than can annexin V [14,15]. In one study, lactadherin bound all activated platelets under conditions where annexin V bound only a small subpopulation[15], suggesting that all activated platelets expose a low level of PS, but that only a subpopulation expose a high level of PS. This high level appears functionally relevant, as coagulation factors, FXa, FVa, FVIII, prothrombin (FII) and FIXa bind specifically to the high annexin V-binding subpopulation [4,16–19]. These annexin V-binding, procoagulant platelets also have depolarized mitochondrial membrane potential (∆Ψm) and inactive integrin α_IIb_β_3_, whereas annexin V-negative activated platelets have normal ∆Ψm and have active integrin α_IIb_β_3_ [4,12,20,21], underlining the distinction between procoagulant platelets and pro-aggregatory platelets.

This apparently all-or-nothing segregation into subpopulations indicates that during activation, platelets **commit** to becoming procoagulant or pro-aggregatory. Although the signaling pathways that control this commitment are not understood, distinct cytosolic Ca^2+^ signals in different subpopulations may play an important role. Cytosolic Ca^2+^ concentration ([Ca^2+^]_cyt_) is a regulator of many platelet actions during thrombosis including PS exposure and release of PS-exposing EVs. Ca^2+^ activates the phospholipid scramblase, TMEM16F (also known as Anoctamin 6 [Ano6]), and inactivates an aminophospholipid flippase [22,23]. This results in net movement of PS to the outer leaflet of the plasma membrane where coagulation factors can bind. PS-exposing EV release requires PS exposure and activation of calpain, a Ca^2+^-dependent protease [24,25], complemented by other, less well-defined pathways [9,26]. Cytosolic Ca^2+^ signaling is therefore central to procoagulant platelet formation. Although platelet granule secretion, α_IIb_β_3_ activation and clot retraction also depend on cytosolic Ca^2+^ signaling, differences in the amplitude or temporal pattern of [Ca^2+^]_cyt_ signals between individual platelets may commit them to form different subpopulations.

Mitochondrial Ca^2+^ signaling also has a central role in procoagulant platelet formation. Opening of the mitochondrial permeability transition pore (PTP) is an important event in procoagulant platelets and is regulated by mitochondrial Ca^2+^ concentration ([Ca^2+^]_mito_). Although less is known about mitochondrial Ca^2+^ signaling, [Ca^2+^]_mito_ signals are also likely to be heterogenous in activated platelets.

In this review, we discuss the interplay between platelet cytosolic and mitochondrial Ca^2+^ signals, highlighting how this might lead to the all-or-nothing commitment to becoming procoagulant, and consider the implications for therapeutic targeting of procoagulant platelets.

## Cytosolic Ca^2+^ Signaling

Most platelet activators trigger a rapid increase in [Ca^2+^]_cyt_ from resting levels <100 nM to up to 1–2 μM. Collagen fibers cluster GPVI, triggering a cascade of protein tyrosine phosphorylation to activate phospholipase Cγ2 [27]. ADP and thromboxane A_2_, released by stimulated platelets, act via Gα_q_-coupled P2Y_12_ and TP receptors, respectively, to activate PLCβ. Thrombin acts via Gα_q_-coupled PAR1 and PAR4, also activating PLCβ. The phospholipases generate inositol 1,4,5 trisphosphate (IP_3_) and diacylglycerol (DAG) from phosphoinositide 4,5 bisphosphate (PIP_2_). IP_3_ opens IP_3_R receptors in the dense tubular system, releasing Ca^2+^ stored there into the cytoplasm[3].

This initial increase in [Ca^2+^]_cyt_ is amplified and sustained by Ca^2+^ entry across the plasma membrane. Ca^2+^ store depletion is detected by STIM1, which activates the channel ORAI1 in the plasma membrane. This process is called ‘store-operated Ca^2+^ entry’ (SOCE) and is a major mechanism for Ca^2+^ entry in platelets [28–31]. Procoagulant platelet formation is inhibited in *Orai1^−/-^* murine platelets or human platelets treated with ORAI1 inhibitors [28,29,32–35]. The nonselective cation channel, TRPC6, also contributes, probably activated by DAG. Na^+^ entry through TRPC6 drives further Ca^2+^ entry via reverse-mode Na^+^-Ca^2+^ exchange (rNCX)[33]. The contribution of TRPC6 is less prominent than that of ORAI1, however, and may be restricted to providing additional Ca^2+^ entry when platelets are coincidentally stimulated by thrombin and collagen-like agonists [32,33]. P2X1, a ligand-gated ion channel activated by extracellular ATP, is a further route for Ca^2+^ entry[36].

Ca^2+^ is removed from the cytosol by transporters and exchangers. SERCA2b and 3 sequester Ca^2+^ into membrane-bound stores. PMCA4b pumps Ca^2+^ across the plasma membrane. NCX removes Ca^2+^ in exchange for extracellular Na^+^, though whether NCX acts in forward-mode to remove Ca^2+^, or reverse-mode to increase [Ca^2+^]_cyt_, depends on the cytosolic Na^+^ concentration[37]. Ca^2+^ is also taken into mitochondria, which will be considered in more detail below.

The interplay between Ca^2+^ release, Ca^2+^ entry, and Ca^2+^ removal produces a wide variety of cytosolic Ca^2+^ signals, including individual transient spikes, irregular trains of spikes (‘ragged spiking’), and sustained increases in [Ca^2+^]_cyt_[38]. Several factors may contribute to the differences between individual platelets. The platelets themselves may be different, with variation in the copy number of receptors and key Ca^2+^ signaling proteins (e.g. IP_3_Rs); the extent of dense tubular system will affect how much stored Ca^2+^ is available for release; and variation in platelet volume may affect the volume of cytosol and hence [Ca^2+^]_cyt_ dynamics[39]. Each of these factors could also differ with platelet age. In addition to platelet-intrinsic factors, different platelets could be exposed to different local concentrations of platelet activators depending, for example, on their proximity to other activated platelets and activators released from them. The heterogeneity of platelet Ca^2+^ signaling is likely to be a complex interplay of many small differences. However, many platelet Ca^2+^ signaling experiments measure the average [Ca^2+^]_cyt_ in whole platelet populations, such as in cuvettes or microplate wells. This approach, although convenient, blurs the dynamics of such signals in individual platelets and the differences between them. Insights from studies of Ca^2+^ signaling in individual platelets (e.g. Refs [38,40,41]) show that there remains a lot to learn about the different patterns of Ca^2+^ signaling and how they are decoded into functional responses.

## Mitochondrial Ca^2+^ Signaling and the Permeability Transition Pore (PTP)

Mitochondrial matrix Ca^2+^ concentration ([Ca^2+^]_mito_) is a key regulator of mitochondrial oxygen consumption and ATP synthesis in many cells, and also regulates procoagulant platelet formation[42]. To enter the matrix, Ca^2+^ must cross the outer and inner mitochondrial membranes (OMM and IMM, respectively). Ca^2+^ readily crosses the OMM through voltage-dependent anion-selective channels (VDAC; despite the name, Ca^2+^ permeates open and closed states of these channels)[43]. Ca^2+^ movement across the IMM is more tightly regulated. The dominant pathway is via the mitochondrial Ca^2+^ uniporter (MCU) complex. The MCU complex consists of the pore-forming unit, MCU itself (CCDC109a), regulators in the IMM such as MCUb (CCDC109b) and EMRE/SMDT1, the latter coupled to the MICU1/MICU2 heterodimer in the intramembrane space [44,45]. The MCU complex is inward-rectifying (toward the mitochondrial matrix), highly Ca^2+^ selective, and with a very large capacity to transport Ca^2+^. The mitochondrial membrane potential (∆Ψm, negative on the matrix side) provides the driving force for Ca^2+^ entry. Despite this driving force, mitochondrial Ca^2+^ entry is limited in cells with low [Ca^2+^]_cyt_ due to inhibition by MICU2. When [Ca^2+^]_cyt_ increases, MICU2 is inhibited and MICU1 is activated, leading to Ca^2+^ entry and an increase in [Ca^2+^]_mito_[46]. Ca^2+^ leaves mitochondrial matrix via a 2 H^+^/Ca^2+^ antiporter (mHCX) and a Na^+^/Ca^2+^ exchange (mNCX). Together, these pathways generate dynamic increases in [Ca^2+^]_mito_ in response to increased [Ca^2+^]_cyt_[47].

Mitochondrial Ca^2+^ uptake is a key step in procoagulant platelet formation. Platelets from *Mcu^−/-^* mice, or human platelets treated with the MCU inhibitor, Ru360, generate fewer procoagulant platelets in response to physiological stimuli without effect on integrin activation[48]. Notably, however, increasing [Ca^2+^]_cyt_ with a Ca^2+^ ionophore bypasses the requirement for MCU[48].

A large rise in [Ca^2+^]_mito_ triggers the **permeability transition pore** (PTP), a large conductance pore of the IMM resulting in depolarization of ∆Ψm and movement of ions and metabolites up to 1.5 kDa[49]. Cyclophilin D (CypD) controls the sensitivity of PTP to [Ca^2+^]_mito_[50]. In the absence of CypD, the threshold [Ca^2+^]_mito_ for PTP opening is much higher. CypD-deficient mouse platelets (*Ppif^−/-^*) generate significantly fewer procoagulant platelets than wild-type in response to physiological agonists[12]. Cyclosporine A (CsA), which inhibits CypD, also inhibits the formation of procoagulant platelets[34]. As with MCU, CypD does not appear necessary for PS exposure in response to high concentrations of Ca^2+^ ionophores, suggesting that mitochondrial Ca^2+^ entry and PTP opening can be bypassed by sufficient high [Ca^2+^]_cyt_. However, in response to physiological stimulation, it is clear the PS exposure and procoagulant platelet formation requires mitochondrial Ca^2+^ entry and PTP opening.

## How Does PTP Regulate Procoagulant Platelet Formation?

Although mitochondrial Ca^2+^ entry is likely to occur in all activated platelets, PTP opening only occurs in platelets that become procoagulant. Panteleev and colleagues proposed an elegant model in which PTP opening is the step that commits platelets to becoming procoagulant [40,51]. In this model, physiological activators trigger an increase in [Ca^2+^]_cyt_ through Ca^2+^ release and Ca^2+^ entry. Some of this Ca^2+^ is taken into mitochondria via MCU leading to an increase in [Ca^2+^]_mito_. However, since cytosolic Ca^2+^ signaling varies between individual platelets, the increase in [Ca^2+^]_mito_ will also vary between individual platelets. In some platelets, [Ca^2+^]_mito_ will be above the threshold for PTP opening. In these platelets, PTP opening commits the platelet to become procoagulant. They lose ∆Ψm (because of PTP opening), expose PS, and inactivate integrin α_IIb_β_3_. In contrast, activated platelets without PTP opening maintain α_IIb_β_3_ activation and are pro-aggregatory. In this model, the Ca^2+^ threshold for PTP opening is therefore the commitment point that converts heterogenous Ca^2+^ signaling into an all-or-nothing response.

How does PTP opening commit a platelet to becoming procoagulant? Specifically, how is this mitochondrial event communicated to TMEM16F in the plasma membrane? We envisage two possibilities: either PTP opening changes cytosolic Ca^2+^ signaling in such a way that it can now activate TMEM16F, or PTP opening releases an additional signal that regulates TMEM16F in addition to cytosolic Ca^2+^ (or, conceivably, removes an inhibitory factor).

Obydennyy and colleagues demonstrated that procoagulant platelet formation was accompanied by a change in cytosolic Ca^2+^ signaling[40]. Thrombin triggered a rapid train of spikes in [Ca^2+^]_cyt_ that varied in frequency and amplitude between individual platelets. However, a transition to high, sustained [Ca^2+^]_cyt_ during procoagulant platelet formation coincided with loss of ∆Ψm and PS exposure. The extent of this high, sustained [Ca^2+^]_cyt_ is difficult to estimate, however, as [Ca^2+^]_cyt_ was mostly measured using Fura Red in these experiments. Fura Red’s fluorescence decreases with Ca^2+^ binding. The high sustained [Ca^2+^]_cyt_ levels that coincide with PTP opening resulted in very low fluorescence. PTP inhibition with CsA reduced the percentage of platelets that became procoagulant but did not affect the thrombin-induced spikes in [Ca^2+^]_cyt_. Similarly, Arachiche et al. also found that Fura Red fluorescence was lower in PS-exposing platelets, and this was also reversed by CsA[52]. These observations suggest that PTP opening causes a further increase in [Ca^2+^]_cyt_, or transition from spiking to sustained cytosolic Ca^2+^ signaling.

In contrast, although Jobe and colleagues found that [Ca^2+^]_cyt_ and [Ca^2+^]_mito_ were higher in procoagulant platelets, they found no difference in cytosolic or mitochondrial Ca^2+^ signaling in CypD-deficient platelets[53]. Similarly, pharmacological inhibition of mitochondrial Ca^2+^ uptake reduced [Ca^2+^]_mito_, ∆Ψm loss and PS exposure, but had no effect on [Ca^2+^]_cyt_. In this study, [Ca^2+^]_cyt_ was measured with Fluo-4, a commonly-used probe with high affinity for Ca^2+^. From these results it appears that PTP opening is downstream of cytosolic and mitochondrial Ca^2+^ signaling, but that PTP opening does not further affect [Ca^2+^]_cyt_, in clear contrast to the conclusions of the studies described above. However, whilst we can readily repeat these results[34], we propose an alternative explanation that reconciles these differing observations.

## A ‘Supramaximal’ Ca^2+^ Signal in Procoagulant Platelets

Recently, we proposed that [Ca^2+^]_cyt_ in procoagulant platelets is much higher than previously appreciated, and much higher than in pro-aggregatory (non-coagulant) platelets[34]. We termed this very high [Ca^2+^]_cyt_ signal a **supramaximal Ca^2+^ signal**, that is, much higher than normally considered maximal. Our observations are based on the use of fluorescent Ca^2+^ probes with different affinities for Ca^2+^. The K_d_ (dissociation constant) of Fluo-4 in vitro is approximately 390 nM. Although the K_d_ will be affected by the intracellular environment, the high affinity of Fluo-4 means that it is likely to be saturated by Ca^2+^ in the low micromolar range. In contrast, Fluo-5N has a very low affinity for Ca^2+^ (K_d_ = 90 μM *in vitro*). This low affinity means that changes [Ca^2+^]_cyt_ in the nanomolar and low micromolar range will have little effect on Ca^2+^ binding to Fluo-5N, whereas [Ca^2+^]_cyt_ of tens of micromolar will increase Ca^2+^ binding and give a fluorescent signal. The fluorescence of platelets loaded with either Fluo-4 or Fluo-5N showed very different patterns in procoagulant and non-coagulant platelets. The Fluo-4 fluorescence in stimulated platelets was much higher than in unstimulated platelets and was similar in procoagulant and non-coagulant platelets. In contrast, in Fluo-5N fluorescence of non-coagulant platelets was similar to unstimulated platelets whereas pro-coagulant platelets had brighter fluorescence. Our interpretation is that [Ca^2+^]_cyt_ in procoagulant platelets is very high compared to non-coagulant platelets. In both subpopulations, [Ca^2+^]_cyt_ is high enough to saturate Fluo-4, which is why there is no apparent difference in Fluo-4 fluorescence between the two subpopulations. This would normally lead to the conclusion that [Ca^2+^]_cyt_ is similar in the two subpopulations. However, although [Ca^2+^]_cyt_ is not high enough in non-coagulant platelets to give a signal with Fluo-5N, [Ca^2+^]_cyt_ is much higher in procoagulant platelets because it is high enough to give a signal with Fluo-5N. The low affinity of Fluo-5N means that [Ca^2+^]_cyt_ must be very high indeed. We estimated [Ca^2+^]_cyt_ as approximately 160 μM.^34^ Although this fairly crude estimate needs further refining, it shows that [Ca^2+^]_cyt_ in procoagulant platelets may be much higher than previously thought.

Very high [Ca^2+^]_cyt_ signals have been reported in other cells. For example, [Ca^2+^]_cyt_ was estimated as greater than 100 μM using low-affinity dyes in ATP-depleted renal proximal tubular cells [54] and MDCK cells[55]. Similarly, in excitotoxic neurons, [Ca^2+^]_cyt_ was estimated as >5 µM by a low-affinity dye but only 0.3–0.4 µM with high-affinity fura-2[56]. Interestingly, these examples are all of cells undergoing cell death. Procoagulant platelets are also necrotic[57], although we have shown that the supramaximal [Ca^2+^]_cyt_ signal in these procoagulant platelets is not simply due to loss of plasma membrane integrity[34].

Supramaximal Ca^2+^ signals provide a means to activate effectors with low Ca^2+^ affinity, including TMEM16F and calpain. The half-maximal Ca^2+^ sensitivity of TMEM16F or phospholipid scramblase activity has been estimated at 10–80 μM in various cells, including platelets, lymphocytes and red blood cells [58–62]. Similarly, calpain, which is needed for release of PS-exposing EVs[24], also requires micromolar [Ca^2+^]_cyt_ for activation [63]. While the Ca^2+^ sensitivity may be altered in cells, with the Ca^2+^ sensitivity of TMEM16F increased by PIP_2_ and PIP_3_, for example[64], supramaximal Ca^2+^ signaling provides a route to specifically activate these low-affinity effectors in procoagulant platelets. However, this does not exclude the possibility that there are other signals that are also released from mitochondria by PTP opening that also regulate PS exposure.

## A Model for Commitment to Becoming Procoagulant

**Figure 1.**
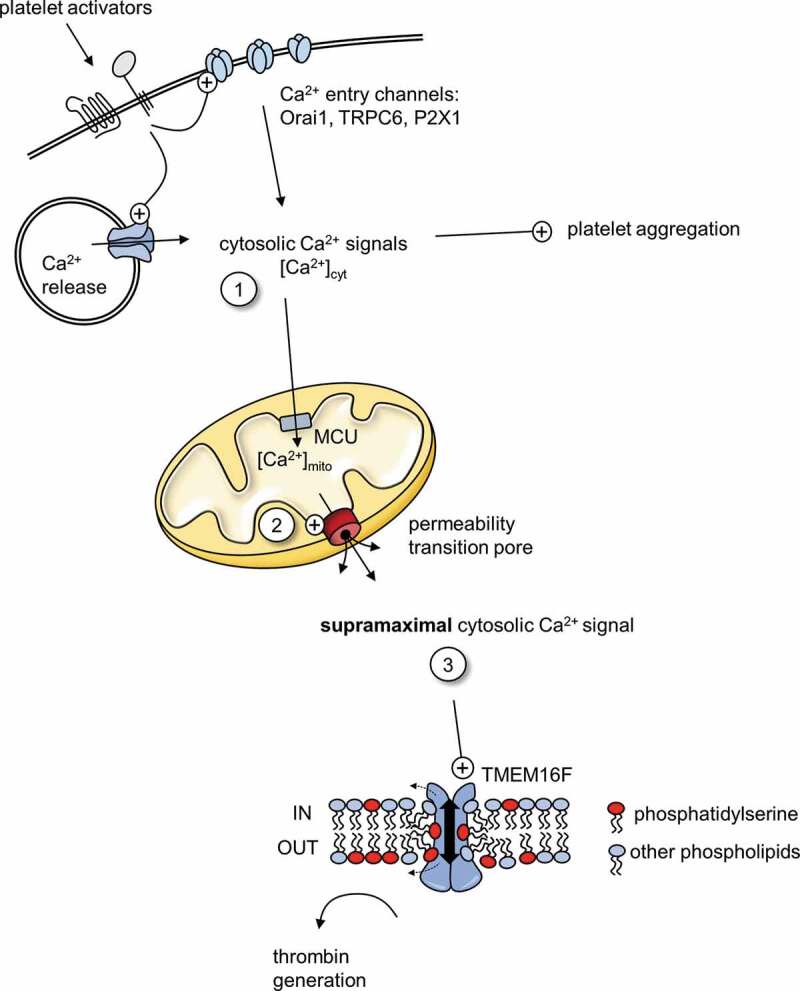
Ca^2+^ signaling in procoagulant platelets

Our model for procoagulant platelet commitment involves three stages of Ca^2+^ signaling (Figure 1). It is based on the model proposed by Panteleev and colleagues [51] but with the addition of supramaximal Ca^2+^ signaling. First, platelet activators such as thrombin and collagen trigger an increase in [Ca^2+^]_cyt_. This occurs through Ca^2+^ release from intracellular stores and Ca^2+^ entry from outside (step 1 in Figure 1). These cytosolic Ca^2+^ signals are sufficient to trigger pro-aggregatory platelet activation in all platelets. However, these signals also vary between platelets, in part due to differences in the expression level of key receptors and Ca^2+^ signaling proteins. Second, some of the cytosolic Ca^2+^ is taken into mitochondria via MCU, which leads to mitochondrial Ca^2+^ signals that vary between platelets. In some platelets, [Ca^2+^]_mito_ increases beyond a threshold such that PTP opening is triggered (2). PTP opening is the commitment to becoming procoagulant, the point of no return. The threshold for PTP opening is reduced by CypD, so when CypD is absent or inhibited, fewer platelets reach this threshold. Third, PTP opening triggers a supramaximal Ca^2+^ signal that activates TMEM16F and leads to PS exposure (3). In contrast, supramaximal Ca^2+^ signaling is not triggered in platelets that do not undergo PTP opening, and these platelets do not become procoagulant but remain pro-aggregatory. A heterogenous initial cytosolic Ca^2+^ is therefore converted into an all-or-nothing response.

Platelet activators, such as collagen and thrombin, trigger a cytosolic Ca^2+^ signal (1) through Ca^2+^ release from intracellular stores and Ca^2+^ entry through plasma membrane channels. The magnitude and temporal nature of these signals varies between platelets. The cytosolic Ca^2+^ signals promote platelet aggregation by regulating granule secretion and integrin α_IIb_β_3_ activation (not shown). Some cytosolic Ca^2+^ is taken into mitochondria via MCU, increasing mitochondrial Ca^2+^ concentration ([Ca^2+^]_mito_). In some platelets, [Ca^2+^]_mito_ exceeds the threshold for permeability transition pore opening (2). This triggers a ‘supramaximal’ cytosolic Ca^2+^ signal in these platelets (3), which are now committed to becoming procoagulant platelets. The supramaximal Ca^2+^ signal activates the TMEM16F, the phospholipid scramblase, resulting in phosphatidylserine exposure on the outer surface of procoagulant platelets.

Our proposed model alters how we might interpret the effect of genetic or pharmacological inhibition on Ca^2+^ signaling in procoagulant platelets. CsA inhibits supramaximal Ca^2+^ signaling measured by low affinity Fluo-5 N, without appearing to affect cytosolic Ca^2+^ signaling measured by high-affinity Fluo-4 [34,53]. We predict that a similar difference will be seen with any pharmacological or genetic approach that blocks mitochondrial Ca^2+^ or PTP opening (steps 2 and 3) but does not affect the initial cytosolic Ca^2+^ signal (step 1). For example, we have seen a similar inhibition of supramaximal Ca^2+^ signaling, with little apparent effect on Fluo-4-measured signals, when mitochondrial Ca^2+^ uptake was inhibited. In contrast, inhibition of Orai1 did inhibit the Fluo-4-measured signals, indicating that Orai1 is involved in the initial cytosolic Ca^2+^ signal[34]. We and others have extensively used high-affinity Ca^2+^ dyes (such as Fluo-4, Fura-2 or similar analogues) to investigate Ca^2+^ signaling during procoagulant platelet formation. In the future, such studies should also include analysis of mitochondrial Ca^2+^ and supramaximal Ca^2+^ signals. Analyzing the variation in pattern of cytosolic Ca^2+^ signaling between individual platelets would also be very informative.

Another source of variation between platelets is in their number of mitochondria. Although we found no significant difference in staining with a fluorescent mitochondrial dye between procoagulant and pro-aggregatory platelets[34], Obydenni and colleagues found that the number of mitochondria in a platelet was a key regulator of procoagulant platelet formation. In this elegant study, platelets with only 1–4 mitochondria were considerably more likely to become procoagulant than platelets with 5 or more mitochondria[65]. This may contribute to the increased procoagulant platelet formation in Wiskott-Aldrich syndrome (WAS), as a greater proportion of platelets had three mitochondria or fewer, although additional mechanisms are also likely to be involved[65]. Fewer mitochondria could mean that more cytosolic Ca^2+^ is taken up by each mitochondrion, increasing the likelihood that [Ca^2+^]_mito_ reaches threshold for PTP opening. Differences in mitochondrial number between healthy platelets could result from small differences in packaging mitochondria into megakaryocyte proplatelets, or release of mitochondria into the circulation[66].

How the supramaximal Ca^2+^ signal is generated is not yet clear. It could be a direct consequence of PTP opening, with Ca^2+^ that has accumulated in the mitochondrial matrix being rapidly released back into the cytosol[50]. PTP opening appears to coincide with a decrease in [Ca^2+^]_mito_ (although this could also be explained by release of the mitochondrial Ca^2+^ dye, Rhod-2, from the mitochondria)[40]. Alternatively, lysis of intracellular organelles may release stored Ca^2+^. Necrosis often involves lysis of intracellular lysosomes, for example[67]. In addition, plasma membrane pores or channels could be involved. Pore-forming executioner proteins are implicated in cell death and could be a route for Ca^2+^ entry, including MLKL in necroptosis[68], gasdermin D in pyroptosis [69,70] and gasdermin E in secondary necrosis[71]. Clarifying the mechanisms that generate the supramaximal Ca^2+^ could identify new anti-thrombotic targets.

## Inhibiting Procoagulant Platelet Formation to Prevent Arterial Thrombosis

Selective inhibition of procoagulant platelet formation, without affecting platelet aggregation, might be a therapeutic approach to prevent arterial thrombosis. In our model, the first step uses the same molecular machinery in procoagulant and pro-aggregatory subpopulations, such as the receptors (e.g., PARs, GPVI) and Ca^2+^ release and inhibition of this step is unlikely to lead to selectivity. There may be some selectivity achieved by inhibiting Ca^2+^ entry channels, such as ORAI1 or TRPC6. Ca^2+^ entry is required to amplify cytosolic Ca^2+^ signaling to a sufficient level to lead to PTP opening, whereas aggregation can take place in the absence of extracellular Ca^2+^. Although procoagulant platelet formation is reduced in *Orai1^−/-^* mouse platelets and with ORAI1 inhibitors [29,32,34,35], there was limited inhibition of platelet aggregation, particularly at higher concentrations of platelet activators [28,72]. However, thrombus formation under arterial shear *in vitro* was reduced, even under non-coagulating conditions, suggesting that platelet aggregation is inhibited. Moreover, loss of ORAI1 in T cells results in severe combined immunodeficiency (SCID)[73], limiting the potential use of ORAI1 blockers as anti-thrombotics.

Ca^2+^ uptake into mitochondria via MCU is another potential target. *Mcu*^−/-^ platelets have reduced procoagulant platelet formation with no apparent effect on aggregation[48]. *Mcu^−/-^* mice are viable and appear grossly normal[74], suggesting that targeting MCU may be relatively safe. Moreover, dysregulation of mitochondrial Ca^2+^ and subsequent mPTP opening also underlies cardiac ischemia-reperfusion injury, making MCU a potential target to limit myocardial injury following arterial thrombosis.

Similarly, PTP inhibition is a potential target. CsA reduces procoagulant platelet formation [12,34]. CsA inhibits CypD and so reduces the Ca^2+^ sensitivity of PTP opening. However, CsA also has other targets, including cyclophilin A, leading to immunosuppression through inhibition of calcineurin, a Ca^2+^-dependent phosphatase. More selective PTP inhibitors might prevent procoagulant platelet formation without immunosuppression.

PTP opening leads to the supramaximal Ca^2+^ signal that we have described. As noted above, better understanding of the mechanism of the supramaximal signal may identify new anti-thrombotic targets, but we are not yet at that stage. At present, the more promising avenues for inhibiting Ca^2+^ signaling in procoagulant platelet formation appear to be to inhibit mitochondrial Ca^2+^ uptake or PTP opening.

Alternatively, platelet procoagulant activity could be directly blocked by inhibiting TMEM16F. Loss of TMEM16F causes Scott Syndrome, a moderate, very rare bleeding disorder[75]. Several *Tmem16f^−/-^* mouse strains have been reported with divergent effects on bleeding time, from no difference to severe bleeding[75]. Inhibition of TMEM16F might therefore be associated with bleeding. However, there are very few reported TMEM16F inhibitors and these are of little use to test whether acute inhibition of TMEM16F increases bleeding risk. R5421 was described as an inhibitor of platelet scramblase activity[76], and though it may directly inhibit TMEM16F with low potency, it has off-target effects on cytosolic Ca^2+^ signaling and on α_IIb_β_3_ activation[77]. Similarly, although epigallocatechin gallate (EGCG), and related polyphenols, are also reported TMEM16F inhibitors [78,79], (though this action has been disputed [80]), these polyphenols inhibit platelet aggregation through a variety of mechanisms [81–86]. Understanding how these molecules affect platelet procoagulant activity could lead to more selective TMEM16F inhibitors in the future.

Finally, procoagulant platelets release PS-exposing EVs, which further promote coagulation. Indeed, EVs contribute approximately 5% of the volume of an arterial thrombi[87]. Their mechanism of release is poorly understood but requires PS exposure and calpain activity [88,89]. Intriguingly, PS-exposing EV release can be inhibited by 2-aminoethoxydiphenylborate (2-APB), a commonly used Ca^2+^ channel modulator, and a related molecule, 3-(diphenylphosphino)-1-propylamine (DP3A). This inhibition was not due to inhibition of PS exposure or calpain activity, indicating that there are as yet unidentified components to the EV release machinery that can be targeted[9].

## Conclusion

The mechanisms that commit a platelet to becoming procoagulant are still incompletely understood. A complex interplay between cytosolic and mitochondrial Ca^2+^ signals is required, leading to permeability transition pore opening and an overload of cytosolic Ca^2+^. In this, procoagulant platelet formation resembles a rapid form of regulated necrosis [57,67,90]. Like necrotic cells, procoagulant platelets may have pro-inflammatory and well as pro-coagulant effects[91]. Inhibiting the formation and functions of procoagulant platelets is a potential therapeutic approach but requires us to further decipher the Ca^2+^ signaling code that commits a platelet to becoming procoagulant.
